# The Hospital Nursing Supplement

**Published:** 1895-01-05

**Authors:** 


"The Hospital" Nursing Mirror.

BEING THE EXTRA NURSING SUPPLEMENT OF "THE HOSPITAL" NEWSPAPER.

[Contributions for this Supplement should be addressed to the Editor, THE HOSPITAL, 428, Strand, London, W.C., and should have the word "Nursing" plainly written in left-hand top corner of the envelope.]

### News from the Nursing World.

### ORGANISED BY PRINCESS BEATRICE.

H.R.H. PRINCESS BEATRICE founded the Berks and Bucks Needlework Guild, which has just supplied 800 garments for distribution this Christmas to institutions and parishes in the two counties to which this well-organised association devotes its services. The beautiful hood which Her Majesty the Queen worked as her own contribution was allotted to the Royal Berkshire Hospital, and keen appreciation of the gift was shown at the meeting recently held on the occasion of its formal presentation. This pretty token of Her Majesty's interest in those for whom the guild works is to be permanently placed in the children's ward. Princess Beatrice's success in this most practical scheme may well encourage other ladies to undertake similar enterprises.

### THE EMPRESS FREDERICK.

THE Empress Frederick, our own Queen's eldest daughter, spent Christmas week in the various hospitals at Berlin, distributing gifts to the patients and holding friendly converse with many a sick man and woman. One institution which she visited was the beautiful memorial of their silver wedding erected by the Empress and her late husband, and dedicated to the use of sick children. Such patients as were able to be up clustered round the royal visitor, who, seated in the middle of a ward, gave to each a Christmas gift. Many could not leave their beds, and to them the Empress afterwards went, speaking tenderly as she presented the eagerly-anticipated toys to the inmates of this pretty children's hospital.

### WESTMINSTER HOSPITAL.

THERE seem obvious advantages in the ward which is set apart for incurables at the Westminster. It is always full, as any occasional vacancy is immediately met by the transfer of a suitable case from a general ward. Unhappily the supply of chronic cases is not likely to fail, so there is no chance of any outsider getting a bed. It is a comfortable thought for those who nurse as well as for the hopelessly afflicted, that one quiet room in the old Westminster Hospital is set apart for those who can never hope to see written down by the doctor "Discharged cured," on their bed tickets.

### THE CAZENOVE PRIZES.

THE family of the Rev. Canon Cazenove, late Governor of Guy's Hospital, have perpetuated his memory in the institution he so dearly loved by endowing certain prizes to be annually competed for by the nursing staff. They will be awarded for the first time this year, each prize-holder becoming entitled to a full course of pharmacy lectures and practical dispensing classes. These advantages are highly esteemed by those who look forward eventually to obtain positions where such knowledge may be essential to success.

### PAST AND PRESENT.

Two stately old houses with wide oaken staircases and handsome ceilings once represented "The Children's Hospital," the first in London to be devoted exclusively to "the little ones." The party walls were partially removed or doors were introduced, and thus wards for poor sick children were evolved from the handsome old rooms. It was a very homely place, in which Dr. West, Mr. Marsh, and other members of the liberal profession devoted their time and skill to the patients. The nurses' accommodation was faulty, and "training" had not come into fashion, but those quaint old houses in Great Ormond Street, with their long strips of garden containing famous trees, were considered a fine quarters for sick children. The structure in which the guests were received at "the children's treat" on Thursday, December 27th, was in every way a contrast to the old building. The present wards have modern advantages in their sanitary walls and polished floors, and the appliances to-day would surprise the nurses of old time. The wards were, indeed, like fairyland with their coloured lamps and trailing greenery, and some of the nurses, neat in person and intelligent of face, compared favourably with those of any institution in the world. Others, alas! conspicuous for rough hair and distorted caps, would not have been tolerated in those days, easy-going as they may now seem, when kindly, untrained gentlewomen voluntarily devoted themselves to the service of the sick and helpless.

### A GOOD YEAR OF HOLLY.

SURELY "a green Christmas" has had a most excellent influence on the birds this year, the holly berries having escaped those voracious little creatures in an almost unprecedented manner. Hence the adornments of the hospitals have benefited, and the wealth of evergreens has been gratefully appreciated. In visiting the different institutions we have observed great variety in design and mode of display, one set of workers owning to "not having done much" in consequence of extra work or fewer helpers, whilst others evidently have had no such restrictions placed upon them. There is a perceptible return to the lavish use of greenery, and a modified display of the muslins and ribbons, which seem a little incongruous, though they will always be popular with young nurses and amateurs. Cotton wool is treated with more respect nowadays than of old, and we have not noticed such reckless waste as used sometimes to vex conscientious custodians of the public funds. Taking them all round, perhaps the hospitals have never looked better than this year, and certainly "Peace on earth, goodwill to men," have been no empty words.

### MIDWIVES.

CANDIDATES already preparing for the January examination of the London Obstetrical Society are placed in some doubt as to the wisdom of going in for it, now that the recent resolution of the Society throws uncertainty on the form of certificate which will be granted. Until the matters at issue between the General Medical Council and the Obstetrical Society are settled, "the formal certificate of the Society will not be immediately forwarded to successful candidates," we are told. The form, and possibly the wording, may be altered, but an examination which has so long been considered a valuable test of proficiency is not likely to lose its public acceptability. Undoubtedly a satisfactory solution of present difficulties will soon be forthcoming, and those who have the best interests of both the public and the midwives at heart will endeavour to secure to the latter those educational advantages which will effectually prevent the mothers of England from falling again into the hands of the ignorant and uncertificated "monthly nurse" of a bygone age.

### PRIVATE NURSES AT SOUTHAMPTON.

The scheme for establishing a private nursing department in connection with the Royal South Hants Infirmary at Southampton was exhaustively discussed at a recent special meeting of the governors. It seems to be considered that such an institution would afford a fair field of employment for the probationers who have received two years' training at the infirmary. The proposed payment of the nurses is not yet made public; the profits to be made out of them obtaining the most prominence at the meeting. This, by the way, was a rather poorly attended one. Nurses who, remaining associated with their own hospital, are assured of board and lodging in sickness, are freed from the anxieties which beset those who work "on their own account." But the ambition of the authorities at Southampton to make a good profit out of the newly-trained nurses whom they profess to wish to help is an undesirable attitude to take nowadays, and is suggestive of the tactics of the private nursing homes which exist solely to increase the incomes of certain speculative ladies. A grant of £500 from the capital of the infirmary is needed to secure a house to accommodate the private nurses, and for this use of the money the consent of the Charity Commissioners must be obtained.

### ISOLATION FOR SUSPICIOUS CASES.

The Dublin Sanitary Association has addressed a letter to the Health Committee of the Local Government Board, as well as to the North and South Dublin Unions, complaining of the want of isolation rooms at the various city dispensaries. Accommodation for patients suspected of infectious disease should certainly under present circumstances be provided, the prevalence of small-pox making it of immediate importance that isolation should, as far as possible, be enforced. No doubt prompt steps will follow the publicity given to this subject by the Sanitary Association.

### QUEEN'S NURSE AT ARBROATH.

The Arbroath District Nursing Association, affiliated with the Queen's Jubilee Institute, has published its third annual report. The number of cases which have been nursed by Miss Forsyth under the doctors in the neighbourhood is sufficient proof of the appreciation which the Queen's Nurse's services have secured there.

### CAGED ORPHANS.

In one of the dormitories at the Church Extension Orphanage at Kilburn an alteration has been introduced since the appearance of the article in The Hospital of December 1st on the subject of "Cages." The iron bar which formerly rendered impossible the unclosing of the doors of the cubicles from the inside during the night has been removed, and each child can now open her own door. In doing so she rings an electric bell in the sister's room adjoining the ward, and thus gives warning of her wakefulness, and possible need of attention. This electric appliance is said to be very costly, and has, therefore, not as yet replaced the old method in all the dormitories. It will probably do so in time as a result of the attention called by us to the drawbacks of the existing system, unless, of course, the total abolition of the cages is enforced.

### AWAITING THE DECISION.

We understand that the report of the Local Government Board inspector on the Lewisham Workhouse Infirmary nursing question has been sent in to the Local Government Board, whose decision may be shortly expected. We have refrained from publishing details as to the inquiry, feeling that it was not well to accentuate differences which were largely personal. The internal government of workhouse infirmaries is, however, a matter of much more than local importance, and it is to be hoped that the decision of the Local Government Board will be such as to place it upon a more satisfactory basis than at present. With new conditions, new rules and regulations become necessary, and the trained nurse and superintendent is a new condition not so completely understood formerly as it is now; the rules regulating the relations of medical superintendent and matron clearly require revision. It would be matter for congratulation if the Board's decision in the present case was to be given simultaneously with the issue of revised regulations.

### A NEW YORK CLUB.

The Home Bureau Registry Club in New York is described as cosmopolitan and unsectarian. It also professes to be only available for members who are graduate nurses of high standing in their profession. The members gave a very successful entertainment early in this month, the club rooms being tastefully decorated on the occasion. A benefit association to aid sick nurses forms part of the organisation of this society.

### SHORT ITEMS.

Mrs. M. Day, of Exeter, has been appointed assistant nurse by the Totnes Board of Guardians.—A movement has been set on foot in Sutherland to secure a trained nurse for each parish; a bazaar will probably be organised to assist the funds.—Only one nurse came up before the London City Board as candidate for the vacant post in the infirmary, and it was reported at the fortnightly meeting that five servants had resigned in the same institution.—The Ladies' Sale of Work at the National Orthopædic Hospital, on November 30th, and December 1st, realised £112 4s., this amount being considerably in excess of that which resulted from a similar undertaking last year.—*Nursing Notes* for January contains an excellent summary of nursing work during the past year, and other interesting matter.—It has been decided to set apart the Hardwicke Hospital for the accommodation of small-pox patients in Dublin.—Thirty pheasants were sent to the Royal Eye Hospital, Southwark, the other day, by H.R.H. the Prince of Wales.—The Countess of Warwick has been elected a member of the Warwick Board of Guardians.—The conference of county ladies over which the Duchess of Bedford presided the other day, advocated the establishment of housekeeping schools in Bedfordshire.

### The Dietetic Management of Infancy.

By George Dickson, M.B., C.M.

### IV.—ATROPHY.

Wasting occurs in a great many of the diseases of children, but wasting in a child unaffected by any obvious disease invariably indicates that his tissues are being starved for lack of nourishment, and it is to such cases that the term "atrophy" is restricted. In ninety-nine cases out of every hundred the fault lies, not in the actual quantity but in the quality of the food supplied, for a child can almost as certainly be starved to death by giving it unsuitable food, as by deliberately withholding from it sufficient food for its needs.

### Treatment.

If, while at the breast, the milk supply appear to be defective, this ought to be supplemented with boiled cow's milk, suitably diluted with barley water, or the child may be weaned altogether and artificial feeding substituted.

If bottle-fed, it should be seen that the general conditions as to dilution of milk, frequency of feeding, &c., are being carried out. If the wasting still continue, one of the infant's foods, such as Mellin's or Nestle's, should be given in addition to the milk.

It should be said at the outset that any form of food selected must have a fair trial, extending over two or three weeks at least, before it is discarded and another substituted. Nothing can be more absurd and useless than the practice of indiscriminately altering the food every two or three days. One of the infant's foods named above will very commonly succeed in counteracting the wasting. Benger's food is also exceedingly valuable in this condition, but should not be given for any great length of time, as, theoretically at least, it must tend to weaken the stomach. It is of exceptional value in tiding over an emergency, but as soon as the child has got the turn a malted food should be substituted instead.

In some cases ordinary cow's milk will not agree, and the child continues to waste in spite of additions. There are quite a number of preparations of milk which may then be tried; sometimes one, sometimes another, will agree.

*Condensed Milk.*—This should not be given for much longer than a month at a time. It contains a large excess of sugar for preservative purposes, and this is apt to occasion flatulence and discomfort. Nevertheless, it sometimes agrees where ordinary cow's milk will not. To begin with, it should not be given stronger than a teaspoonful to a teacupful of warm water, or, better still, barley water. This concentration can be increased up to one in four, if well tolerated.

*Lœfflund's Rindermilch* is a condensed milk in which sugar is not the preservative agent employed, and it is said to be an exceedingly useful preparation.

*Artificial Human Milk* is so prepared from cow's milk as to approximately correspond to human milk in composition. The character of the curd, however, is not altered in the process, and it still coagulates in cheesy masses. It is a convenient preparation, requiring, as it does, no dilution and no trouble beyond heating, and it should always be tried when other preparations fail.

*Strippings.*—A food highly commended by Eustace Smith is the milk obtained by remilking the cow after the ordinary daily supply has been withdrawn. This form of milk, called "strippings," is rich in cream but poor in curd. It generally agrees well given diluted with two-thirds of water or barley water.

*Peptonised Milk.*—In cases where all other forms of milk fail, peptonised milk will generally be tolerated, and will produce a temporary rally. As the child improves the amount of peptonisation should be gradually reduced, by lessening the amount of the digestive agent added, and by shortening the time during which it is left in contact with the milk.

In all cases where any of the above are given a return should very gradually be made to good cow's milk, where possible, beginning with small quantities such as one or two teaspoonfuls in half a bottle of food and cautiously increasing.

*Intolerance of Cow's Milk.*—In some very rare instances cow's milk, however diluted and prepared, seems to disagree, and in such cases whey and cream or whey and barley water should be given.

| | |
|---|---|
| Whey two tablespoonfuls, | Whey two tablespoonfuls, |
| Barley water two tablespnfls., | Water two tablespoonfuls, |
| Cream one tablespoonful. | Cream one tablespoonful. |

If the child improve on either of the above, milk should very cautiously be added, beginning with a teaspoonful to each meal. For such children Cheadle recommends "bread jelly food," prepared as follows: Take a thick slice of wheaten bread (bread made from seconds flour) and soak it in water for six or eight hours; then squeeze all the water out of it, place it in fresh water and boil for one and a half hours. The gruel thus made should be strained, rubbed through a fine hair sieve and allowed to cool, when it settles into a jelly. Of this jelly about a tablespoonful should be added to eight ounces of water to make the food of the consistence of cream. This preparation of itself is quite inadequate as a food, but it forms a harmless diluent, to which supplementary articles can be added. Dr. Cheadle recommends the addition of raw meat juice (see below) and cream in the following proportions: Bread jelly food, five tablespoonfuls; raw meat juice, six teaspoonfuls; cream, two teaspoonfuls. As the child improves the meat juice should be increased at the expense of the bread jelly. Bread jelly food probably possesses few advantages over the simple and harmless barley water. To this meat juice and cream can be added as to the bread jelly to make it sufficiently nutritious as a food.

In cases of extreme debility and emaciation a child should not be allowed to suck from a bottle, but should be fed with a teaspoon, small quantities being given very frequently, even a teaspoonful every ten or fifteen minutes where there is great weakness. Raw meat, whether in form of juice pulp or Caffyn's Liquor Carnis is a food of the very greatest value for weakly babies. The albumen, in its uncoagulated condition, is much more digestible than that of cooked meat, and appears to be more nutritious. The juice, which is more convenient than the pulp, is prepared as follows: Mince the best steak very fine, add water in the proportion of one of water to four of meat. Stir, and allow to stand cold for one hour. The juice should then be forcibly expressed through muslin by twisting it. Pulp is prepared by scraping the meat with a knife, or by pounding it after mincing in a mortar, and then rubbing it through a sieve. It is most suited to children of ten or twelve months old, and upwards. Of either of the above as much as two ounces may be given in the twenty-four hours, or even more. Of Caffyn's Liquor Carnis the dose is half to one teaspoonful every four hours. The raw meat preparations may be mixed with the food that is being given to the infant, but in cases of great debility and weakness they should be given alone with a teaspoon. Children, as a rule, seem rather to like them than otherwise, but if they show any repugnance the preparation may be mixed with a little chocolate, sweetened with sugar, or otherwise disguised in such a manner as will suggest itself. As raw meat very quickly decomposes, care should be taken that the meat is fresh. It should be prepared at least twice a day.

### Wants and Workers.

Is there any home in or near London which for £20 per annum would take an incurable lady of 70 years of age, helpless through rheumatism? Her small income excludes her from most homes, and is not sufficient inducement for private persons to give her the attention she needs?—Address *Helpless.*

### The American Woman at Home.

By Our Own Correspondent.

### V.—HER LOVERS.

It can hardly be denied that even the most dress-loving woman does not dress for herself alone. It is done *pro bono publico*, perhaps, so far as conscious intent goes, only to make other women envious, but that is founded on a deeper, more instinctive sentiment, to be found pleasing in men's eyes. If this is the law of the sex all the world over it is not less so in America than elsewhere, although there woman treats man in a more cavalier fashion than anywhere else on the habitable globe. Remarkable is the combination of the American woman's sentiments on the subject—the absolute need for man in the mass, and the cool contempt for any individual of the sex.

Certainly it is difficult for a man to gain a romantic interest in an American girl's eyes. She has been used to attention from her cradle. She has had flowers and candy, photographs and gifts from a score of lovers before, in the ordinary sense, she goes out into society at all. You can hardly call her a flirt, because the conventions of her country make flirting a matter of course. It is not to be supposed that there are no conventions across the Atlantic, and that every woman can do that which is right in her own eyes; but the limits of American propriety are so different from ours that English eyes may sometimes fail to see them. In the larger cities severer notions of the proprieties are coming in, but the mass of the people hold to the old free ways. A girl may receive letters from a man who is not her accepted lover, and her mother, though she may fret and fidget to know their contents, does not feel that she has the right to ask. If a girl meets a young man whom she regards as agreeable she asks him to call on her. There is nothing unmaidenly in the invitation; it implies no more than the admission that an occasional chat with him would be pleasant. It would be regarded as infinitely more forward if he asked permission to visit her, and, as she shrewdly argues, she can exercise a judicious selection in her visitors much more effectively and with more courtesy by herself taking the initiative in invitation than if she left it to the man. To solve the difficulty by leaving the selection of male visitors to her mother does not occur to anybody. It isn't the mother whom it concerns. When our young gentleman calls he makes no pretence of asking for Mr. or Mrs. So-and-So; it is mademoiselle he wants to see, and her he asks for. She is not even bound, unless she so chooses, to introduced him to her family circle; it is quite open to her to receive him alone, her parents will not venture to intrude, and the only interruption the *tête à-tête* is liable to is the arrival of another admirer. On the strength of such an acquaintance as this he may ask the young lady to go to the theatre with him; to go out driving with him, even in the evening; to accept his escort, and his alone, at any and every time. He is not only permitted but expected to offer her flowers and sweets; only at gifts of pronounced value will she draw the line as compromising herself by their acceptance. Anything short of that means nothing, and if he is weak enough to think otherwise and let his heart get entangled, he is not expected to complain if it is refused.

Of course, he may be accepted, but then an engagement more or less matters so little to the girl. Mamma, watchful and wary, cannot, does not, attempt to prevent the betrothal, but usually succeeds in keeping back the announcement if the youth is a detrimental, and then—a visit to a watering-place, a tour in Europe, the advent of another admirer or two, and the thing is at an end. Often enough the end is easily reached, for with the uncertainty and pleasant excitement of the pre-proposal stage the young lady's interest in the man ceases. "All she loved was love," the lover was a mere lay figure in the affair, and any other will do as well as he. Some day, perhaps, she will find someone whom she will treat less lightly, but perhaps not, and when she marries her husband will be as much the subject of her caprice as any of her lovers.

"An American woman," said an English resident in New York, "regards her husband as the man who supplies her with diamonds, and he is content to be so regarded," which struck the Englishman as strange. In the Christmas number of a popular woman's journal, the editor begs his readers not to demand gifts of jewellery from their mankind in this year of commercial depression. "Many a business man," says the writer, "can trace his downfall to the diamond earrings for which wife or daughter begged so hard." The wife and daughter who work such mischief are not wilfully unkind, consciously unreasonable, but it has never entered their minds that the husband or father is anything but a sort of jackal. They leave him for months, for years at a time, to slave at business and live at hotels while they spend his money in Europe. The better type of woman does not, indeed, act so selfishly, the woman who loves her husband, who shares his anxieties and sympathises with his struggles; but the American woman who has such feelings is a higher type than the Englishwoman whose acts are the same as hers, for it is all a matter of grace, not of duty. Jonathan receives as a privilege what John takes as a matter of course.

What, then, is the fundamental relation of the American man to the American woman? On this point one may safely generalise. If a woman is content to tyrannise over the superficial side of a man's life without inquiring into its more serious depths, she will get just what she demands and no more. She will get bouquets, dresses, diamonds, to the full extent of his purse or beyond; but she will not get that companionship which a true-hearted woman, as she outlives the days of her vanity, will value more. Nay, if she has given a false and shallow ideal of womanhood to her husband, he will continue to follow it when with her faded cheeks she can no longer fulfil it. One could not but know why some of the middle-aged women dressed so gaily, forgetting that youth and youth's charm cannot be simulated by youthful dress, and that a woman who in the passage of the years has not established a deeper claim to her husband's loyalty than was won by her girlish bloom, is little likely to retain it. It is, after all, so much easier to win a man than to keep him. The American girl has opportunities for the first beyond any on the face of the earth; but after marriage life is founded on the same basis for all women, and I do not think that on the latter point either her instincts or her training give her any advantage over others.

### Appointments.

Croydon General Hospital.—Miss Mary Bird has been appointed by the unanimous vote of the committee to the Matronship of their hospital, and takes up her duties in February. She was trained at Worcester Infirmary, and afterwards held responsible positions there. Miss Bird came to Croydon fifteen months ago as Matron of the Croydon Nurses' Institute, where she has since done excellent work. She carries with her to her new post the good wishes of a large circle of friends.

Reynard Cottage Hospital.—Miss Evelyn Hurlbutt, who received her training at Guy's Hospital, has been appointed Matron of the Reynard Hospital. We congratulate her on her appointment.

### Everybody's Opinion.

[Correspondence on all subjects is invited, but we cannot in any way be responsible for the opinions expressed by our correspondents. No communications can be entertained if the name and address of the correspondent is not given, or unless one side of the paper only be written on.]

### AN APPROPRIATE THANK-OFFERING.

"Nurse A. E. C." writes: Please accept the accompanying flannel night-gowns for your Christmas distribution of clothing. They are sent by a grateful member of the Royal National Pension Fund for Nurses and Sick Pay Policy-holder. It is the first needlework done after recovery from a long illness, and is a small thank-offering for renewed health. The sick pay has been the chief source of support, which I acknowledge with heartfelt gratitude to the founder of a Fund which is an inestimable boon to nurses.

[Three beautifully-made and very warm night-gowns accompanied this note, and we cordially congratulate "Nurse A. E. C." on the practical form of her thank-offering.—Ed. *T.H.*]

### A POLICY-HOLDER'S TRIBUTE.

"Nurse T. L" writes: As the old year closes my thoughts turn with thankful gratitude to all connected with the Pension Fund. Their kindness in paying my premiums has been great, and has saved me worry and anxiety. They will, I believe, continue their bounty for two years more, when I shall attain the age of 55, at which time my pension commences. The comfort of knowing that old age is provided for is, indeed, unspeakably great. If it had not been for the wisdom and forethought of the founder of the Fund, I and many more would only have the workhouse as our last home. At present I own one room, very comfortably furnished. Everything I have was left me by a dear old lady with whom I lived as nurse-companion for about twenty-one years, and who died in her ninetieth year. Accept my earnest good wishes for the health and happiness and prosperity of each and all connected with our Fund.

### CORRECTION.

Miss Pauline Peter writes: I am very glad to hear that the Wolverhampton District Nurses have now a "Home" set apart for their use.

### Christmas in Germany.

(Communicated.)

Christmas Eve is the time round which the thoughts of the Germans centre, Christmas Day itself being of comparatively small account. I was staying in a large country house in Mecklenburg, where I had been nursing a "Frau Generalin," and there I witnessed preparations for keeping Christmas on a grand scale. Everyone had been busy for the past month in sewing, knitting, and embroidering, and the forms that the work took filled my English mind with awe and wonder—awe at the thousands of minute stitches, and wonder at their being expended on such inferior objects and materials, for the commonest of towels had intricate borders embroidered upon them, and the poorest of ticking was made into bags for dried peas, beans, &c., to hang in the storeroom; not plain bags by any means, but things decorated with an immense amount of embroidery, surmounted by the cypher and the all-pervading coronet. These works take much time, so the women folk were stitching from morning till night, Sundays included! Indeed, a present that has not been "worked" is looked at askance if the donor is a woman. Men give strange things—hair-brushes (not with silver backs!), collars and cuffs, umbrellas, soap, and other things of undoubted utility, for the Germans, including the nobles, are nothing if not practical.

Then the cooking! The making of our English plum puddings and mince pies is trifling when compared with their gingerbread, marchpane, cakes, and endless queer-looking sweetmeats. The gingerbread is the only thing about which any tradition still clings, spices of all kinds being used to commemorate the frankincense and myrrh. The "cradle" shape of our correctly-made mince pies has, however, given place in Germany to rounds and balls. And then at last Christmas Eve comes, and everyone sighs with relief at the preparations being finished.

The presents are arranged on tables round the room, each person having their own table, and in the middle of the room stands the tree, gay with tapers and coloured glass balls—a far better plan, it seems to me, than that of tying everything on to the tree and then as laboriously cutting all off again.

Even the English nurse was provided with a table by these kindly Germans. They greatly pitied me for never having seen a proper Christmas before; it was useless to protest that in England, too, very proper Christmases are often celebrated; this was merely regarded as a flight of imagination, similar to a rash assertion that England was not always foggy. After the "tree" then comes the feasting, which generally lasts for a week — the boars' heads, geese, and baby calves, roasted whole, making one fancy oneself back in the Middle Ages. Every meal is enormous, and the wine "flows," the only word which expresses the way in which it is dispensed.

There was service in the church (to which we went with rugs and foot muffs) on Christmas Day; but the impression which a German Christmas leaves on the mind is one of feasting and present giving.

### Presentations.

On Christmas Eve the nurses of the Blackheath and Richmond Institution presented Mrs. Smith and Miss Duncan with a pair of handsome carvers. To Miss Plumbe, matron of the Richmond Branch, the nurses gave a chased silver frame.

On Christmas Day the nursing staff of the St. Helena Home, London, presented Miss Robertson, the Lady Superintendent, with a silver table lamp and an ebony and silver tea tray, as a mark of esteem and gratitude.

On her departure from Hitchin, where she has been head of the Diocesan Nursing Institution, Miss Sawyer was presented with a silver tea pot, cream jug, and a purse containing five pounds from "her grateful friends in St. Mary's Parish."

On Christmas morning the staff of the Hove Hospital, Portslade, presented the Matron, Miss Mawer, with a silver breakfast cruet.

A handsome afternoon tea table was presented to Miss Ridley, the Matron of the Hospital for Epilepsy and Paralysis, Regent's Park, on Christmas Day, by the Nurses.

The Matron (Miss Baynes) of the Isolation Hospital, Norwich, was, on Christmas Day, the recipient of a handsomely-fitted dressing case, with engraved monogram, a token of affection and esteem from the nurses.

### Minor Appointment.

Bury Infirmary.—Miss Edith Milnes, who was trained at the Huddersfield Infirmary and spent three years there, has been appointed Charge Nurse of the [illegible] ward of thirty beds at the Infirmary, Bury, and takes [illegible] good wishes for success with her to her new work.

### Entertainments.

AT ST. THOMAS'S HOSPITAL gifts, music, and decorated wards made Christmas Day bright to the patients, charming combinations of colours being brought into play in various parts of the building. In one fine ward a delicious shade of yellow prevailed, the gracefully-draped pillars and the shaded lamps and hanging lanterns making a most harmonious whole. Delicate sprays of ivy outlined some of the pale yellow hangings with good effect. Red of all shades and delicate greens were carried out in such completeness that the grand wards were transfigured in a manner difficult to describe, but harder still to forget. No wonder the patients lay in quiet content watching the charming scenes. On the 26th the annual carol singing took place, the Nightingale Probationers, under the guidance of the Home Sister, singing several carols in each of the wards. From Westminster Bridge the whole hospital made an attractive evening picture, the glow of the many-coloured lights streaming out through the long windows.

The entertainments in GUY'S HOSPITAL have been very numerous this year. Each day in the octave of Christmas has been marked by concerts in some of the wards, a "tree" for the children and a magic lantern display being also provided on the 29th. On Christmas Day the nigger troupe went round the hospital, and good as their annual performance has always been, the students are considered to have this season surpassed all previous displays. During the preliminary lighting up of the tree in the duly decorated ward (Martha) a procession of residents and students arrived on the scene. Each carried a quaint little bundle of humanity (such wee creatures some of them), imported from other wards to share in the Christmas tree distribution of toys. Each small patient was warmly enveloped in a snowy miniature blanket, and a bright-coloured flannel gown and pretty pinafore completed the attractive outfit.

At the LONDON HOSPITAL the ward entertainments are concentrated on Christmas Day itself. The nurses sing carols in the early morning, and after breakfast "Father Christmas" generally makes his appearance with other seasonable guests, such as carol singers from distant parts of London. Tea, an early and a lengthy meal, was prettily served and greatly enjoyed. Men by no means scorn the good tea and coffee, but the women linger longest over it. The entertainments given in each ward during the evening did enormous credit to everybody. The students had devoted much time and talent to preparation, and in the tall "ladies" who took the female characters in the various shows it was difficult to recognise the grave young doctors of ordinary working days. The decorations were universally pretty and seasonable, but in very few of the busy wards were they elaborate. The admirable condition of the appliances and the orderly appearance of each portion of this vast establishment provoked much admiration. The children's entertainment took place on the last day of the old year, and, as on former occasions, it proved a brilliant success. Punch and Judy formed the first item on the programme, and as soon as the performances were concluded the tapers were lighted on the fine trees which were as usual given by Mr. Robert Barclay, and Mr. Leopold Rothschild again repeated his handsome annual donation of £10, a cheque for £20 was sent by an anonymous donor, and Madame Giorgi forwarded £5. Cheques were also received from Mrs. Couper, Mrs. Lowe, Mrs. Dorling, Miss Barrow, and other kind friends. The supply of toys was excellent, some being sent by H.R.H. the Duchess of York, the Editor of *Truth*, Mrs. Cobb, Mr. J. H. Crossman, jun., "The Christmas Fairies," by Miss Violet Charles, Mrs. Goodwin Stevenson, Mrs. Ralph, Mrs. Robert Prior, Miss Ida Pitt, Lady Trevelyan, Miss Nix, Mrs. H. White, Mrs. Underhill, Miss Silk, Miss Edith Mulley, and others. Amongst the guests were: The Duchess of Bedford, Mrs. Couper, Mr. and Mrs. John Henry Buxton, Mr. and Mrs. John Hampton Hale and party, Mr. and Mrs. Cobb, Lieut.-General Sir William Stirling Hamilton, Bart., R.A., Colonel the Hon. W. Le Poer Trench, General Sir John Watson, K.C.B., V.C., Mr. and Mrs. Frederick Green, Mr. P. M. Martineau, the Rev. S. A. Thompson Yates, Mr. and Mrs. Jesser Coope, the Rev. Father Gorman, Dr. Stephen Mackenzie, Dr. Sansom, Dr. and Mrs. Gilbart Smith, Dr. and Mrs. Frederick Smith, Dr. and Mrs. Percy Kidd, Dr. Giddings, Dr. Schorstein, Dr. Dawson, Mr. and Mrs. Openshaw, Dr. and Mrs. Herman, Miss Gordon, Miss Davidson, Miss Nott-Bower, Miss Moir, Miss R. Paget, Miss Power, Miss Coleman, Miss Honnor Morten, Misses Tindal, Mrs. Basil Martineau, Mrs. Isaacs, Miss de Pledge, and others. A large number of former students, sisters and nurses were also conspicuous amongst the crowds of guests who inspected the hospital and nursing home at the conclusion of the entertainment.

A dinner, consisting of the usual Christmas fare, was provided on the 25th for the patients at the CITY OF LONDON HOSPITAL, VICTORIA PARK, some members of the committee of management and governors being present on the occasion. Christmas cards, books, and periodicals were distributed, and the dolls and toys provided by *Truth* and other friends of the institution were given to the juvenile patients. In the evening a musical entertainment took place, which was much appreciated by all present.

Turkeys and port wine were in evidence at the HOSPITAL FOR CONSUMPTION AT BROMPTON on Christmas Day, both supplied, according to custom, by liberal friends of the institution. A Christmas tree and concert on the 28th ult. formed a successful entertainment, a substantial parcel of presents being received by each patient.

The annual Christmas entertainment at the EAST LONDON CHILDREN'S HOSPITAL, SHADWELL, took place on Saturday; when Punch and Judy and two fine Christmas trees gave much pleasure to patients and visitors.

A most successful ball in aid of the NEW HOSPITAL FOR WOMEN IN EUSTON ROAD was recently held at Lincoln's Inn Hall, 1,100 guests being present on the occasion.

AN enjoyable Christmas was spent at the HOSPITAL FOR EPILEPSY AND PARALYSIS, Regent's Park, a fine turkey being sent, as in previous years, by a former patient. Recitations were given by Miss Matilda Ellis, and the Matron, Miss Miriam Ridley, and her staff devoted themselves to entertaining the patients.

The entrance hall at the CHELSEA INFIRMARY is by no means a bare-looking spot at any time, but just now it is transformed by evergreens into general prettiness which forms a fitting entrance into wards which are at present very gay. Each of these has its own colour, curtains, flowers, and shades being all arranged with admirable taste. The inmates must wonder at their novel surroundings, and the nurses deserve warm congratulations on the results of their labours. The poor little children look very cosy in their turkey red frocks, with white pinafores, kept in place by sashes of the same material as their frocks, and they have their share of toys too; the white quilts look so fresh and pretty that the rate-supported laundry which provides them may well be envied by less-favoured institutions. The children's party, provided by *Truth's* sixpences, and the gay Christmas tree, which had been the centre of attraction in the ward during the week, formed a fitting New Year's Day entertainment.

At the CENTRAL LONDON SICK ASYLUM, CLEVELAND STREET, on December 27th, the annual distribution of certificates was made to the nurses, who, at the completion of their training, atisfied the examiner. Mr. Hobbs, chairman of the board, expressed pleasure at the advance that had been made by systematic training of the nurses. An enjoyable evening was afterwards spent by the nurses and their friends.

An excellent entertainment, consisting of tableaux and music, took place last week at the Edinburgh Royal Infirmary, the resident medical staff being the organisers and performers. A crowded audience keenly appreciated the successful entertainment given in the great kitchen, which, according to custom, was transformed for the occasion.

The Bradford Children's Hospital was made very gay with decorations, and a happy Christmas Day was spent by the little patients.

At Bradford Infirmary Christmas Day was ushered in with carols sung by the choir of St. John's Church, and later in the day the prize choir of the Eastbrook Chapel entertained the inmates with hymns and carols. Christmas trees were provided in the children's wards, and a festival dinner for everybody was supplied by the generosity of the Mayor.

At the Eye and Ear Hospital the patients were also in debted to the Mayor of Bradford for an excellent Christmas dinner, and this institution, like the infirmary, was decorated with considerable taste.

The inmates of Mill Road Infirmary, Liverpool, were greeted by carols on Christmas morning, sung in the wards by the nurses; and a merry morning was spent preparing for the great event of Christmas dinner, fully appreciated by all able to partake of it. Afterwards the convalescent patients were allowed to visit the other wards of the hospital, a piano being provided in each block. The wards were tastefully decorated with mottos and evergreens. After tea entertainments were held in the different wards by a party of minstrels, who generously volunteered their services, and gave a varied programme in each ward.

Christmas passed very pleasantly at the Essex and Colchester Hospital, which was very prettily decorated for the festival. A substantial tea was provided, and on the following day a concert was much enjoyed by the patients.

The pupils of the Portsmouth High School for Girls gave their annual Christmas tree and entertainment to the sick children and adults in Portsmouth Hospital on December 15th. The wards were excellently decorated and the gifts and amusements highly appreciated.

On December 27th the annual Christmas tree and entertainment provided by the Ladies' Committee was held at Huddersfield Infirmary in the large accident ward, when a large number of visitors were present. The Mayor, who presided, distributed to patients, nurses, and servants useful gifts provided by the ladies. To the latter a hearty vote of thanks was proposed and seconded by the patients at the conclusion of an excellent miscellaneous entertainment. The patients were regaled with an abundance of Christmas fare. On the following evening a supper and dance for nurses and servants took place, a handsome meal being provided in the board-room.

On Christmas Day, as on previous occasions, the wards and corridors of the Horton Infirmary, Banbury, were prettily decorated by the matron, nurses, convalescent patients, and porters. Mr. K. Hadland sent a Christmas letter and card to each patient, and at half-past twelve dinner was served. All patients who were well enough partook of Christmas fare, followed by dessert and wine, the gift of Mr. C. H. Davids. The Rev. Graham Jones visited the wards during the afternoon with the boys of Christ's Church choir, who delighted the patients by carols and hymns. Each patient received a useful present as a souvenir of "Christmas Day at the Horton Infirmary."

Christmas festivities commenced early at Caterham Asylum, the usual weekly dance being held on Tuesday, the 18th, and on the following Friday an interesting entertainment being given by Mr. Davidson. On Christmas Eve the asylum band performed several suitable selections in the front hall, where they were heard by most of the inmates. On Christmas Day, at eleven a.m., a service was held in the chapel, prettily decorated for the occasion by Mrs. Warren (the matron), Miss Hinman (her assistant), and Miss Coe (one of the head attendants). The wards presented a charming appearance, some of the nurses displaying great artistic taste. In the infirmaries warm, red rugs lay across the foot of the beds. The patients dined at a quarter-past one p.m., and thoroughly appreciated their roast reef, plum pudding, and liberal dessert. The nurses' dinner consisted of turkey, duck, and other dainties. In the evening the patients were entertained by the nurses, there being a piano in almost every ward and plenty of music and singing going on. Some of the patients decked themselves out in paper crowns, aprons, and caps out of the bon-bons provided by the charge nurses. The medical superintendent, Dr. Elliot, and his assistants, Dr. Campbell and Dr. Thomas, were untiring in promoting the entertainments throughout the building; and the steward, Mr. Schilling, was greatly complimented on having proved such an able caterer for the whole establishment.

One of the most deserving Dublin charities is the Coombe Lying-in Hospital, Dublin, and its many friends and patrons will be pleased to learn that it is in a flourishing condition. Christmas Day was a bright and pleasant one to all within the walls; the decorations were charming, and Miss Egan and her staff nurses were indefatigable in amusing the patients. In the evening an impromptu concert was successfully organized by the nursing staff. Lord Ardilaun and Mr. Lombard, governors of the hospital, sent flowers and greenery for the decoration of the wards.

A special fête was organized for the benefit of the inmates of Temple Street Children's Hospital, Dublin, by the Ladies Committee. The halls, corridors, and staircases were gaily decorated, and in each of the three wards stood a large Christmas tree. The sisters of the Sacred Heart Convent, Roscrea, sent a jacket to each of the sixty child-patients, as well as special Christmas boxes. The committee were busy from half-past three to six p.m. distributing toys and other gifts. Afterwards the rev. mother and sisters entertained the Archbishop, Bishop of Canea, Lord Mayor and Lady Mayoress, the committee, the medical staff and their families, and numerous other visitors.

Under the auspices of Surgeon-Colonel Greene and Surgeon-Lieutenant Greene, the detachment of the Army Medical Staff Corps, stationed at Arb.ur Hill Station Hospital, Dublin, celebrated Christmas in a worthy manner. The Board of Works gave evergreens from Phœnix Park for the decorations of the reading-room; and photographs of Field-Marshall Lord Wolseley and General Moncrieff were hung in prominent positions. In the evening a concert was held.

A promenade concert, in aid of the Orthopædic Hospital, Brunswick Street, took place at the Ancient Concert Rooms, and was in every respect such a brilliant success, that it is proposed to repeat the entertainment.

On Christmas afternoon, at the "Sailor's Rest," Dublin, a social tea meeting was held, at which over eighty people were present, seventy being sailors of different nationalities, a large number belonging to the cross-channel steamers which had been out in the recent gale. All the guests greatly appreciated the magic-lantern views shown, and the carols, &c., sung by the lady and gentlemen visitors.

A choir selected from the nursing staff, Rotunda Hospital, led and trained by Miss St. Clair, of St. Laurence's Home, rendered some quaint old carols very sweetly for the patients and visitors on Christmas Day. The wards were beautifully decorated by the students and nurses of this fine old maternity hospital. The new wing, a very handsome structure, will shortly be ready for occupation. Evergreens for decorating purposes were sent by the Lord Lieutenant, Lady Harrel, and others; whilst turkeys for the patients' dinners were given by Mrs. Egan, Sister Lucy, and Nurse Loane. A Christmas Tree was supplied by Mr. Ramsay.

Dr. Drury, in a letter to the editor of the *Irish Times*, mentions that he had just handed to Miss Huxley, Lady Superintendent of Sir Patrick Dun's Hospital, the sum of £5 13s. 6d. collected on Christmas Eve by a party of ladies and gentlemen whilst singing carols in Terenure Road, Orwell Road, Highfield Road, and Dartry Park.

